# Resveratrol promotes myogenesis and hypertrophy in murine myoblasts

**DOI:** 10.1186/1479-5876-11-310

**Published:** 2013-12-13

**Authors:** Anna Montesano, Livio Luzi, Pamela Senesi, Nausicaa Mazzocchi, Ileana Terruzzi

**Affiliations:** 1 Department of Biomedical Sciences for Health, University of Milan, Milan, Italy; 2 Metabolism Research Centre and Department of Endocrinology and Metabolic Diseases, San Donato Hospital and Scientific Institute, Milan, Italy; 3 Division of Metabolic and Cardiovascular Sciences, Metabolism, Nutrigenomics and Cellular Differentiation Unit, DIBIT-San Raffaele Scientific Institute, Milan, Italy

**Keywords:** Resveratrol, Proliferation, Myoblast, Differentiation, Myocyte, Myogenic Regulatory Factors, Myotube, Hypertrophy

## Abstract

**Background:**

Nutrigenomics elucidate the ability of bioactive food components to influence gene expression, protein synthesis, degradation and post-translational modifications.

Resveratrol (RSV), natural polyphenol found in grapes and in other fruits, has a plethora of health benefits in a variety of human diseases: cardio- and neuroprotection, immune regulation, cancer chemoprevention, DNA repair, prevention of mitochondrial disorder, avoidance of obesity-related diseases. In skeletal muscle, RSV acts on protein catabolism and muscle function, conferring resistance against oxidative stress, injury and cell death, but its action mechanisms and protein targets in myogenesis process are not completely known. Myogenesis is a dynamic multistep process regulated by Myogenic Regulator Factors (MRFs), responsible of the commitment of myogenic cell into skeletal muscle: mononucleated undifferentiated myoblasts break free from cell cycle, elongate and fuse to form multinucleated myotubes. Skeletal muscle hypertrophy can be defined as a result of an increase in the size of pre-existing skeletal muscle fibers accompanied by increased protein synthesis, mainly regulated by Insulin Like Growth Factor 1 (IGF-1), PI3-K/AKT signaling pathways.

Aim of this work was the study of RSV effects on proliferation, differentiation process and hypertrophy in C2C12 murine cells.

**Methods:**

To study proliferative phase, cells were incubated in growth medium with/without RSV (0.1 or 25 μM) until reaching sub confluence condition (24, 48, 72 h). To examine differentiation, at 70% confluence, cells were transferred in differentiation medium both with/without RSV (0.1 or 25 μM) for 24, 48, 72, 96 hours. After 72 hours of differentiation, the genesis of hypertrophy in neo-formed myotubes was analyzed.

**Results:**

Data showed that RSV regulates cell cycle exit and induces C2C12 muscle differentiation. Furthermore, RSV might control MRFs and muscle-specific proteins synthesis. In late differentiation, RSV has positive effects on hypertrophy: RSV stimulates IGF-1 signaling pathway, in particular AKT and ERK 1/2 protein activation, AMPK protein level and induces hypertrophic morphological changes in neo-formed myotubes modulating cytoskeletal proteins expression.

**Conclusions:**

RSV might control cell cycle promoting myogenesis and hypertrophy *in vitro*, opening a novel field of application of RSV in clinical conditions characterized by chronic functional and morphological muscle impairment.

## Background

### Skeletal muscle differentiation

**Figure 1:**
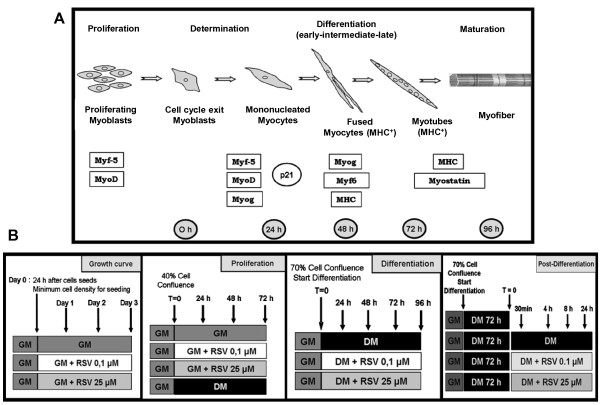
**Stages of myogenesis and experimental study design. A)** Schematic illustration of skeletal muscle differentiation. **B)** Description of each experimental phase of the study protocol.

Skeletal muscle differentiation is a dynamic multistep process that involves two simultaneous phenomena. The first is the induction of muscle-specific genes expression by Myogenic Regulatory Factors (MRFs), such as Myf-5, MyoD, Myf-6 and Myogenin [1-8] (Figure 1A).

The second phase is the commitment of myogenic cells into skeletal muscle cells: mononucleated undifferentiated myoblasts break free from the cell cycle, cease to divide, elongate and fuse into multinucleated myotubes [1-3,9-12] (Figure 1A). A differentiation marker in neo-formed myotubes is the transcription induction of structural muscle-specific genes, such as Myosin Heavy Chain (MyHC), the major structural protein in myotubes [9-11].

At the molecular level, several positive and negative cell cycle regulators have been identified. Progression through cell cycle phases is dependent on consecutive activation and inhibition of phosphoproteins by cyclin-dependent kinases (CDKs) complexed with their activators cyclins [1,4,5,13].

Furthermore, cytoskeletal reorganization occurs before and after myoblast fusion: a number of studies indicate that N-Cadherin (N-cad), a member of calcium-dependent cell adhesion molecules, and Alpha-Sarcomeric Actinin (α-act), an actin binding protein, have a central role in these cytoskeletal reorganizations [14,15].

Further, AMP-activated protein kinase (AMPK) appears to act as a master regulator of skeletal muscle metabolism and as a negative feedback control to maintain muscle hypertrophy [16].

When the cellular AMP/ATP ratio is high, AMPK is activated, inhibiting ATP-consuming anabolic pathways and promoting ATP-producing catabolic pathways: as result protein synthesis and cell growth are suppressed [16-18].

### Muscle hypertrophy

Skeletal muscle is a dynamic tissue that can either increase or decrease its mass in response to a variety of environmental causes such as exercise, nutrients and starvation.

Two major signaling pathways have been identified that control these processes through two distinct positive and negative mechanisms respectively, mediated by either Insulin Like Growth Factor 1 (IGF-1) or Growth and Differentiation Factor (GDF8), otherwise known as Myostatin [19,20].

Skeletal muscle hypertrophy can be defined as an overall increase of muscle mass, as a result of an enlargement of the size of pre-existing skeletal muscle fibers accompanied by enhanced protein synthesis without an apparent increase in the number of myofibers [19-21].

Insulin Growth Factor-1 (IGF-1) is among the best characterized muscle growth promoting factors. Mainly produced in the liver under the control of the Growth Hormone (GH), its expression is located also in the skeletal muscle, suggesting a paracrine/autocrine role of IGF-1 in positively regulating muscle growth. IGF-1 acts through direct interaction with its own receptor IGF-1 R, a tyrosine-kinase leading to the final activation of AKT by the generation of phosphatidylinositol-3,4,5-triphosphates (PIP3) [20,22-24].

Many studies have established that IGF-1 strongly activates muscle hypertrophy by stimulating the PI3-Kinase/AKT pathway. IGF can activate any of the three AKT isoforms, and currently both AKT1 and AKT2 have been implicated in myogenesis. Protein levels of AKT1 remains constant from proliferating to differentiating cells, whereas the levels and activity of AKT2 increase with differentiation [25]. AKT, in turn, activates the downstream kinase mTOR, which stimulates p70 S6 kinase and other effectors, ultimately culminating in enhanced protein synthesis [22-24].

### Resveratrol properties

Resveratrol (RSV) (3,5,4′-trihydroxystillbene) belongs to the huge group of polyphenols found naturally in a variety of plants, especially in the peel of grapes and peanuts. RSV has received important attention because of a number of reports highlighting its benefits *in vitro* and *in vivo* in a variety of human disease, including cardio- and neuroprotection, immune regulation, cancer chemoprevention, DNA repair, Sirtuins activation, prevention of mitochondrial disorder, avoidance of obesity-related diseases [26-40].

The versatility of RSV lies in its diverse targeting of membrane and intracellular receptors, signaling molecules, biogenesis enzymes, oxidative systems, DNA-repair mechanisms and transcription factors, as well as in the wide range of possible RSV-induced effect, including cellular proliferation, cell-cycle arrest, differentiation and cell death [26,27,29].

To elucidate the underlying mechanism of RSV action, much research has been focused on different tissues and cell types such as myocardial cells and hepatocytes [30-32,37]. But, since RSV has been shown to act on skeletal muscle metabolism and function [41-45], less attention has been given to its effects on myogenesis [46].

### In vitro model for myogenesis study

C2C12 murine immortalized cell line provides a good *in vitro* model for the study of the major steps of myoblasts proliferation and differentiation [6,47-50].

In this cellular model, undifferentiated myoblasts are recognizable as flat, fusiform or star-shaped cells, which appeared scattered on the substrate and rigorously mononucleated. After reaching confluence or 24 hour after serum removal, C2C12 cells are considered myoblasts in an early differentiation stage and they are characterized by changes in myoblasts orientation, lengthening and thickening. Later, confluent mononucleated myocytes begin to fuse forming multinucleated myotubes (intermediate differentiation), positive for the characteristic muscle-specific protein MyHC. Myotubes become wider and longer over the next few days as additional myocytes fusion. Multinucleated and large myotubes appear to form a network with numerous nuclei arranged in multiple linear arrays (late differentiation) (Figure 1A).

In the present work we investigated potential mechanisms mediating the effects of two different doses of Resveratrol (0.1 μM and 25 μM) on cell cycle regulation, skeletal muscle differentiation and during the genesis of hypertrophy in C2C12 myoblastic cells (Figure 1B).

## Methods

### Materials

Mouse C2C12 myoblastic cells were purchased from the European Collection of Animal Cell Cultures (ECACC). Reagents were purchased from Sigma Chem. (St. Louis, MO, U.S.A.). Primary antibodies: anti-MyoD (C-20), anti-Myf-5 (C-20), anti-Akt1/2 (N-9), anti-MyHC (H-300), anti-p21 (C-19), anti-Myogenin (D-10), anti-Calnexin (H-70), anti-GDF-8 (N-19), anti-IGF-1 (G-17), anti-N-Cadherin (H-63), anti-p120 (H-90), anti-AMPKα1/2 (H-300), anti-pERK1/2 (E-4), anti-ERK1 (K-23), anti-ERK2 (C-14), anti-p53 (FL-393) monoclonal or polyclonal primary antibodies and the peroxidase-conjugated or rhodamine-conjugated secondary antibodies were purchased from Santa Cruz Biotechnology (Santa Cruz, CA, U.S.A.). Alpha-Sarcomeric Actinin primary antibody was purchased from Sigma Chem. Co. (St. Louis, MO, USA). Anti-phospho-Akt (Ser 473)(D9E) and phospho-AMPKα (Thr 172) (40H9) were purchased from Cell Signaling Technology (Danvers, MA, U.S.A.).

In particular, Resveratrol was purchased from Sigma Chem. (St. Louis, MO, U.S.A.) and, according to the manufacturer’s instruction, it was dissolved in sterile water.

### Experimental procedures

C2C12 cells were maintained at 37°C in humidified 5% CO2 atmosphere in a growth medium containing DMEM (Dulbecco Modified Eagle Medium) supplemented with 20% (v/v) FBS (Fetal Bovine Serum), 1% penicillin-streptomycin and 1% L-glutamine up to 70% confluence.

During proliferation phase, cells, seeded at 6 × 10^2^ cells/cm^2^, were maintained in mitogen-rich growth medium (GM) as single myoblasts. These proliferating cells were treated with RSV 0.1 and 25 μM. These two doses represent the optimal concentrations to induce effects on differentiation process without any significant toxicity for cells [42,46]. This observation was validated by our growth curve and cell viability test.

According to RSV half-life, medium was changed every 8 hours.

Mouse myoblast C2C12 immortalized cell line is a subclone of C2 myoblasts, which spontaneously fuse and differentiate into multinucleated myotubes as a result of both the achievement of myoblast confluence (as in the case of cells seeded in growth medium with 20% FBS = GM) and the removal of the serum growth factors (as for cells transferred in DMEM supplemented with 1% HS, Horse Serum = DM) [6,47-50]. Figure 1B explains experimental study design in each phase of the protocol, with cell confluence percentage, treatments start time and duration.

RSV action was evaluated by Real-Time-PCR, Western Blot and Immunofluorescence analysis during proliferation phase and in the induction, progression and termination of myogenesis. RSV effects on hypertrophy process were also studied.

### Growth curve and cell viability test

To study RSV action on C2C12 myoblast proliferation, we performed growth curve assay as described [51].

C2C12 myoblasts were plated in 60 mm × 15 mm culture dishes at 40% confluence and grown in GM with or without RSV (0.1 and 25 μM). Medium was changed every 24 h and the experiment lasted until control cells achieved 70% of confluence (3 days) (Figure 1B).

Every day, the cells were trypsinized and stained with trypan blue. Both viable (non-stained) and non-viable (blue) cells were counted using a hemacytometer. The total cell count average values for each single day were used to plot a growth curve for myoblasts treated with RSV (0.1 and 25 μM) and control (GM and DM). Cell viability was calculated by dividing the non-stained viable cell count by the total cell count.

In addition, every day morphological changes were examined.

### Real-Time-PCR (RT-PCR) array analysis

RT^2^-PCR Array plates produced by SABiosciences (SABiosciences Corporation, Frederick, MD 21703 USA) were utilized to simultaneously analyze the expression levels of a panel of genes.

We studied the following genes expression during proliferation phase (24 h): Cyclin A2, Cyclin B1, Cyclin C, Cyclin D1, Cyclin E1 and Cyclin F, using Mouse Cell Cycle RT^2^ Profiler™ PCR Array, as described [52].

Total RNA was isolated from C2C12 using the RNeasy Plus Mini Qiagen Kit (Qiagen GmbH, Germany). Total RNA (1 μg) was reverse transcribed using RT^2^ First Strand Kit (SABiosciences Corporation, Frederick, MD 21703 USA). The reverse transcripts were used as templates for analysis of gene expression level using RT^2^ – PCR Arrays plates according to the manufacturer’s instructions. Each sample was run in triplicate. The expression level of the housekeeping genes chosen for normalization in the threshold cycle (Ct) for each experimental conditions and then the fold-change (ΔΔCt) for each gene from treated group compared to the control group (GM control time 0), was calculated. If the ΔΔCt is greater than 1, the result may be reported as a fold up-regulation. If the ΔΔCt is less than 1, the result may be reported as a fold down-regulation.

### Electrophoretic techniques and immunoblotting analysis

C2C12 myofibers were homogenized in lysis buffer (50 mM Tris/HCl, pH 7.4, 150 mM NaCl, 1% Triton X-100, 1 mM sodium orthovanadate (Na_3_VO_4_), 1 mM EDTA, 1 mM PMSF, 1 mg/ml aprotinin, 1 mg/ml leupeptin, 1 mg/ml pepstatin) and shaked for 1 h at 4°C. Detergent-insoluble material was removed from the cell suspension by centrifugation at 12,000 × g for 30 min. Proteins content was quantified using Bradford method. Aliquots of 30 μg supernatant proteins from the different samples were resolved by SDS-PAGE. Electrophoresed proteins were transferred to nitrocellulose membrane (Protran®, Whatman® Schleicher & Schuell) as described [53]. The membranes were incubated with specific antibodies and then incubated with HRP-coniugated anti species-specific secondary antibodies. Immunoreactive bands were visualized by an enhanced chemiluminescence method (Amersham Pharmacia Biotech, Piscataway, NJ, USA) The membrane was stripped and reprobed with an antibody α-calnexin to confirm equal protein loading per sample.

Quantitative measurement of immunoreactive bands was performed by densitometric analysis using the Scion image software (Scion Corporation, Frederick, MD, USA).

Data were then presented as fold change (FC) of the control.

### Immunofluorescence analysis

For indirect immunofluorescence, C2C12 cells were fixed in 4% paraformaldehyde, permeabilized with 0.2% Triton X-100, and blocked with PBS containing 1% bovine serum albumin. Cells were then immunostained with specific antibodies rhodamine-conjugated (Santa Cruz Biotechnology, California, USA) and nuclei revealed with DAPI staining. Cells were observed using fluorescence Leica DM IRE2 microscopy and Nikon Eclipse 50I microscopy and images of myotubes were captured using respectively IM50 software and Nis-Elements D 4.00 software (Leica Microsystems, Switzerland and Nikon Instruments Europe BV, Netherlands) for size comparison. Data were displayed and analyzed using Adobe® Photoshop® CS4.

For myotubes length and diameter size, the average measurement on each slide was generated from approximately 150 myotubes. 10 fields were randomly chosen and all MyHC-positive multinucleated cells containing at least 3 nuclei in each field were measured. The data were then converted to percentage increase of the control (DM). To quantify the differentiation and fusion of C2C12 cells after treatments, we calculated the fusion index as the average number of nuclei in of MyHC-positive multinucleated cells above total nuclei. In the same way, the data were then converted to percentage increase of the control (DM).

### Statistical analysis

All experiments were performed three times. For array, immunoblotting and Immunofluorescence analysis, statistical evaluations were performed by t-test. Data are presented as the mean ± SD. Results were considered statistically significant if p ≤ 0.05.

## Results

### Proliferative phase

In proliferative phase, we investigated MRFs protein synthesis and morphologic features in C2C12 cells after exposure to 0.1 or 25 μM of RSV for different time periods (Figure 1B). We used a control in which RSV was not added to the medium (GM or DM).

We first examined RSV action on C2C12 proliferation rate. Every day, growth time and morphologic feature changes of C2C12 were evaluated.

**Figure 2:**
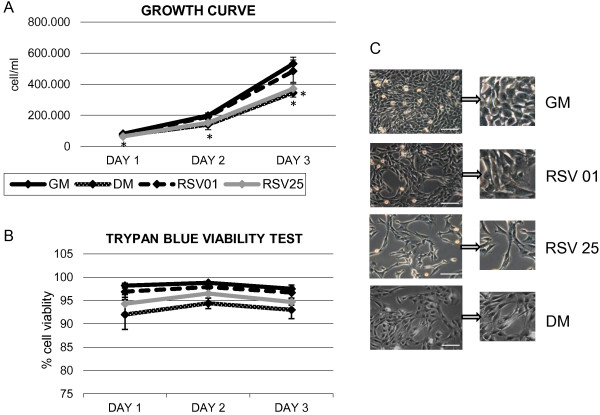
**Growth curve, viability test and morphological study. A)** C2C12 proliferation rate through kinetics of cell growth curve. RSV treatment induces a decrease of cell division in respect to untreated control cells. Significance: * p ≤ 0.05. **B)** C2C12 viability assay shows the absence of cell mortality in all treatment conditions. **C)** Phase contrast images, collected at day 3 of proliferation curve, illustrate the morphological changes observed in RSV-treated cells. The cells lose their characteristic circular shape to achieve a new elongate morphology in respect to control cells (GM). Interesting to note the analogy in cell morphology between 25 μM RSV and differentiation control (DM). Scale bar 200 μm.

Proliferation curve, in Figure 2A, showed that RSV treatment induced a decrease of cell division with respect to untreated control cells (GM). This effect was dose-dependent: RSV 0.1 μM had a minimal effect, comparable to untreated cells, while the highest concentration, RSV 25 μM, showed an important action on proliferation control. (Day 1: DM vs GM p ≤ 0.05; Day 2: DM vs GM p ≤ 0.05. Day 3: DM vs GM p ≤ 0.05, RSV 25 μM vs GM p ≤ 0.05).

In Figure 2B, viability assay graph showed the absence of cell mortality in all treatment conditions.

A very important support to those data were the morphological changes observed in cells treated with 25 μM of RSV: the cells seem to lose their characteristic circular shape, typical of the active proliferation phase, to achieve a new elongated morphology. Phase contrast images, collected at day 3 of growth curve, confirmed those morphological features (Figure 2C): morphological changes in cell size and shape are compared in detail, emphasizing the analogy between DM cells (differentiation control) and 25 μM RSV-treated cells.

**Figure 3:**
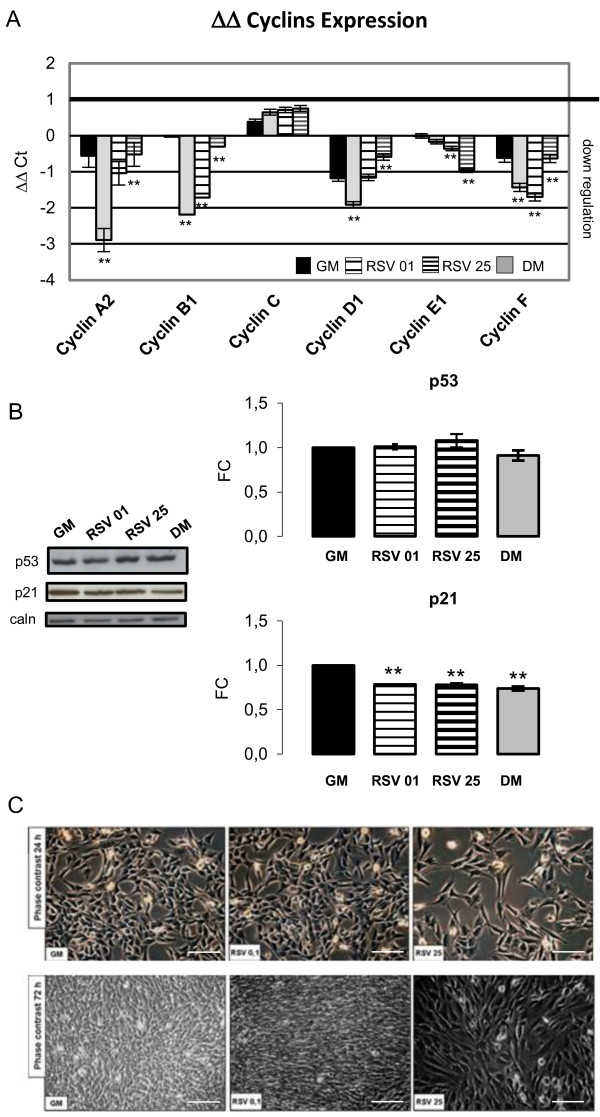
**RSV action on cell cycle regulation in proliferation phase. A)** Real-Time-PCR during proliferation phase (24 h), proves an actual decrease in Cyclins expression levels after treatment with RSV, in a similar way to DM condition, in respect to GM time 0 condition. Significance: * p ≤ 0.05; ** p ≤ 0.01. **B)** p53 Western Blot analysis, during proliferation phase, shows how RSV treatment does not modify p53 protein amount in respect to GM control condition. p21 Western Blot analysis reveals a significant decrease in protein content in both 0.1 and 25 μM RSV-treated cells, following DM trend, in respect to growth control GM. Significance: * p ≤ 0.05; ** p ≤ 0.01. Representative immunoblots of analyzed proteins are shown. **C)** Phase contrast images collected at 24 and 72 h of proliferative phase show morphological changes mentioned in Figure 2. Scale bar 200 μm.

Most Cyclins expression seems to decrease with the onset of differentiation, when cells are blocked in G1 phase [1]. To achieve additional confirmation of data obtained from the growth curve, viability test and morphological studies, we performed quantitative Real-Time-PCR during proliferation phase (24 h), to prove an actual decrease in Cyclins expression levels (Figure 3A). As shown in the panel, RSV treatments cause a significantly down-regulation in Cyclins expression, following DM control condition, in respect to GM time 0 control (Cyclin A2: DM vs GM T0 p ≤ 0.01; RSV 25 μM vs GM T0 p ≤ 0.01. Cyclin B1: DM, RSV 0.1, RSV 25 μM vs GM T0 p ≤ 0.01. Cyclin D1: DM vs GM T0 p ≤ 0.01; RSV 25 μM vs GM T0 p ≤ 0.01. Cyclin E1: RSV 0.1 μM vs GM T0 p ≤ 0.01; RSV 25 μM vs GM T0 p ≤ 0.01. Cyclin F: DM, RSV 0.1, RSV 25 μM vs GM T0 p ≤ 0.01).

To verify the absence of RSV cytotoxic effects on C2C12, we evaluated in Western Blot analysis the protein levels of the apoptotic marker p53 [54] during proliferation phase (Figure 3B), showing how RSV treatment does not modify p53 protein amount in respect to GM control condition. Phase contrast images in Figure 3C, collected at 24 h and 72 h of proliferative phase, illustrated the morphological changes in RSV-treated cells with respect to control.

Furthermore, to corroborate RSV action on cell cycle regulation, we measured the protein content of cell cycle regulator p21 during proliferative phase. RSV treatment (both 0.1 and 25 μM) seems to cause a significant decrease in p21 protein levels with respect to control (DM vs GM p ≤0.01; RSV 0.1 vs GM μM p ≤0.01; RSV 25 μM vs GM p ≤0.01) (Figure 3B). The lower protein content in RSV-treated cells with respect to growth control (GM) is comparable to differentiation control cells (DM). Since p21 promotes cell cycle exit and induces cellular differentiation [5,55,56], we might suppose that RSV could induce cell cycle arrest and differentiation.

To investigate RSV action on differentiation induction, we determinated protein amount of two early MRFs: MyoD and Myf-5, key markers of differentiation induction [1-8].

**Figure 4:**
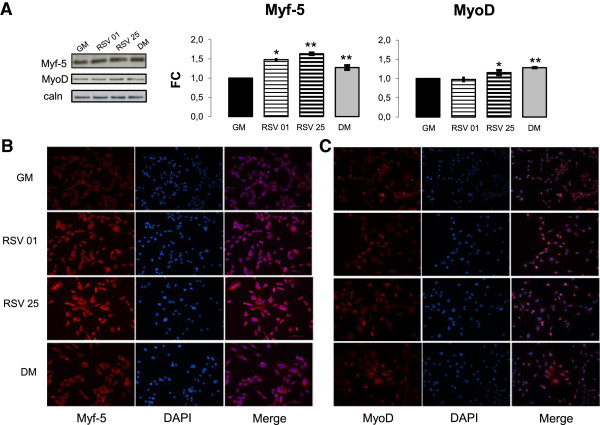
**RSV action on early MRFs protein expression during proliferation phase. A)** Protein levels of early MRF Myf-5 significantly rise in RSV treatments (both 0.1 and 25 μM) and DM condition compared with GM. Instead, Western Blot assay indicates that only 25 μM RSV treatment significantly increases early MRF MyoD protein amount, following DM condition. Significance: * p ≤ 0.05; ** p ≤ 0.01. Representative immunoblots of analyzed proteins are shown. **B, C)** Immunofluorescence images confirms Western Blot results: RSV treatments promotes early MRFs protein expression and morphological features cited above. Scale bar 50 μm.

Figure 4A elucidated the significant increase of Myf-5 and MyoD protein levels after RSV stimulation (Myf-5: DM vs GM p ≤ 0.01; RSV 25 μM vs GM p ≤ 0.01; RSV 0.1 vs GM p ≤ 0.05) (MyoD: DM vs GM p ≤ 0.01; RSV 25 μM vs GM p ≤ 0.05).

In addition, we studied morphological changes in myoblasts through MyoD and Myf-5 Immunofluorescence analysis during proliferative phase (24 h, Figure 4B-C). Knowing that MyoD and Myf-5 represent important markers for early myogenesis stage and regulates skeletal muscle commitment [6,7], these results prove that RSV can advance differentiation induction.

The absence of resveratrol cytotoxicity, associated with a decrease in mRNA expression of most important Cyclins, a decrement of proliferation rate and morphological changes coupled with a significant rise in early MRFs protein expression led us to suppose that this polyphenol could promote differentiation induction through cell cycle control.

RSV seems to be able to direct the acquisition of a specific myogenic phenotype: from undifferentiated myoblasts to myocites [2,8].

### Differentiation induction and progression

Sequential expression of MRFs at a specific stage is pivotally important for the success of the myogenesis [1-3,6,8,11,12].

To study differentiation induction and progression, we analyzed protein levels of main MRFs and skeletal proteins during early (24 h), intermediate (48–72 h) and late (96 h) differentiation by Western Blot.

**Figure 5:**
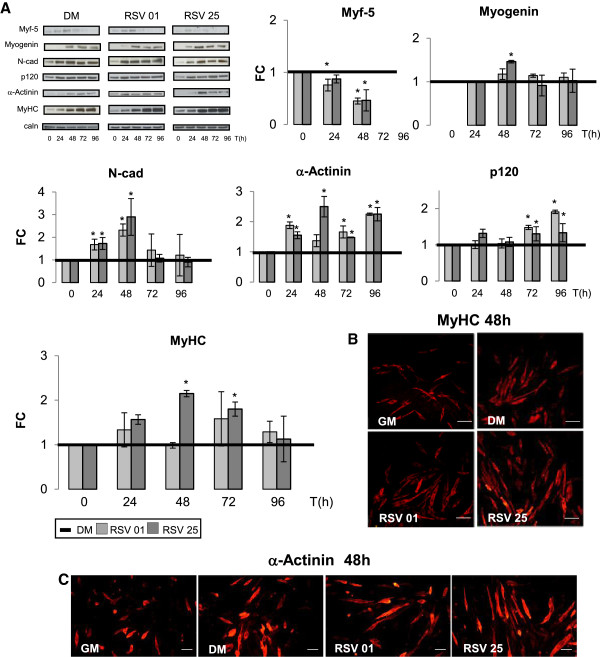
**RSV role on differentiation phases and cytoskeletal protein. A)** RSV treatment seems to cause an anticipate decrease of early MRF Myf-5 in respect to DM control. Myogenin protein content confirms how both 0.1 and 25 μM RSV treatments could anticipate the MRFs expression in respect to DM. RSV reveals an imperative action on protein content rise of key structural proteins like N-Cadherin, Alpha-Sarcomeric Actinin and p120. MyHC blot shows how RSV causes an important increase in MyHC protein levels corroborate to DM. Significance: *p ≤ 0.05; **p ≤ 0.01. Representative immunoblots of analyzed proteins are shown. **B, C)** MyHC and Alpha Actinin Sarcomeric immunofluorescence images collected at 48 h of differentiation show a major density of cells positive for these two structural proteins in RSV conditions in respect to control conditions. Scale bar 200 μm.

Figure 5A shows Myf-5 protein levels during differentiation phases: in RSV-treated cells protein content of this early MRFs decreased during differentiation progression until it appeared undetectable (72 h). Instead, in DM condition Myf-5 protein levels diminished but more slowly than in RSV-treated cells and at 72 hours are still detectable (RSV 0.1 μM vs DM 24 h p ≤ 0.05; RSV 0.1 vs DM 48 h p ≤ 0.05; RSV 25 μM vs DM 48 h p ≤ 0.05). RSV treatment might anticipate the protein expression of early MRFs.

Myogenin protein levels, in Figure 5A, confirmed how both 0.1 and 25 μM RSV treatments could advance the expression of early MRFs in respect to DM control, promoting differentiation progression (RSV 25 μM vs DM 48 h p ≤ 0.05).

For myotubes to form, fusion-competent myoblasts need to migrate towards each other or towards existing myotubes, align and establish close cell-cell contacts so that membranes can fuse [57-59]. N-Cadherin is of utmost importance in this process [14,60]. RSV revealed an imperative action on protein levels of key structural proteins N-Cadherin, p120 Catenin, associated with M-Cadherin activity, and Alpha-Sarcomeric Actinin proteins. Blot in Figure 5A elucidates this effect: during all differentiation stages, RSV treatment significantly increased protein content of specific skeletal proteins responsible of neo-myotubes formation (N-Cadherin: RSV 0.1 μM vs DM 24 and 48 h p ≤ 0.05; RSV 25 μM vs DM 24 and 48 h p ≤ 0.05); (p120: RSV 0.1 μM vs DM 72 and 96 h p ≤ 0.05; RSV 25 μM vs DM 72 and 96 h p ≤ 0.05); (Alpha-Sarcomeric Actinin: RSV 0.1 μM vs DM 24,72,96 h p ≤ 0.05; RSV 25 μM vs DM 24,48,72,96 h p ≤ 0.05).

Graph in Figure 5A illustrates 0.1 μM and 25 μM RSV effects on MyHC protein expression during all differentiation phases. In particular, 25 μM RSV caused an important increase in MyHC protein content in respect to DM (RSV 25 μM vs DM 48 and 72 h p ≤ 0.05).

Immunofluorescence analysis after 48 hours of differentiation (Figure 5B-C) provided an additional prove of RSV role in differentiation progress: images of MyHC (Figure 5B) and Alpha-Sarcomeric Actinin (Figure 5C) protein expression showed a difference in the number of cells positive for these two structural proteins. Moreover, Figure 5B-C illustrates the highest density of MyHC and Alpha-Sarcomeric Actinin positive cells in 25 μM RSV-treated cells in respect to DM. In RSV conditions cells became more elongated and assumed a bipolar morphology, showing the presence of early myoblasts clusters, in respect to control.

**Figure 6:**
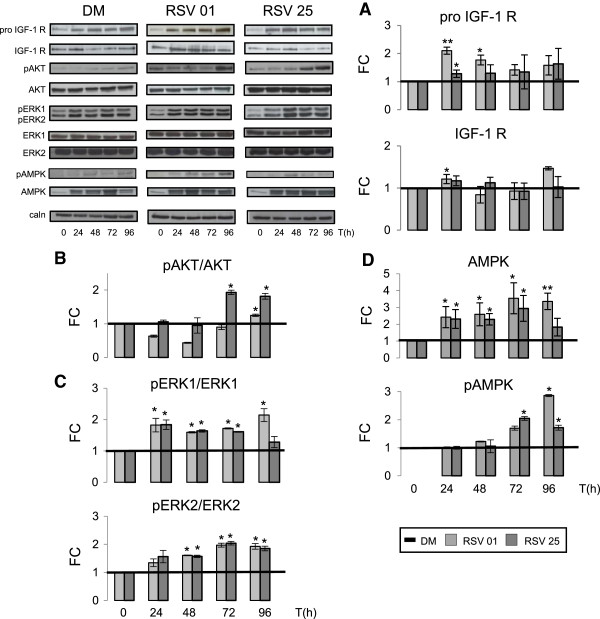
**RSV action on IGF and ERKs signaling pathways during differentiation phases. A)** Western Blot analysis shows how RSV treatments cause a tendency to increase levels of Pro-IGF-1 R protein and IGF-1 R protein during differentiation. **B)** In the last phase of differentiation, RSV promotes AKT activation. **C)** Furthermore, RSV stimuli enhances ERK pathways in all differentiation phases. **D)** As shown in the panel, RSV seems to cause a significant improve in AMPK protein content during all phase of differentiation and, in the end of differentiation, RSV also promotes AMPK activation. Significance: * p ≤ 0.05; ** p ≤ 0.01. Representative immunoblots of analyzed proteins are shown.

IGF-1 represents the major anabolic factor in skeletal muscle, promoting mitogenic and anabolic effects through the activation of the AKT signaling pathway. Its biological activity requires its binding to a specific receptor (IGF-1 R) [61,62]. IGF-1 R is synthesized as a single polypeptide chain (Pro IGF-1 R) that is processed to mature receptor. As shown in Figure 6A, RSV caused a tendency to increase levels of Pro-IGF-1 R protein and IGF-1 R protein during all analyzed differentiation time (Pro-IGF-1 R: RSV 0.1 μM vs DM 24 h p ≤ 0.01; 48 h p ≤ 0.05; RSV 25 μM vs DM 24 h p ≤ 0.05) (IGF-1 R: RSV 0.1 μM vs DM 24 h p ≤ 0.05).

As expected, RSV stimuli increases the phosphorylation state representing activated AKT (Figure 6B): in particular, RSV 0.1 μM at 96 h of differentiation and RSV 25 μM at 72 and 96 h after differentiation induction (pAKT/AKT: RSV 0.1 μM vs DM 96 h p ≤ 0.05; RSV μM vs DM 72, 96 h p ≤ 0.05).

Widely described in literature is the important role of ERK 1/2 MAP kinases signaling in muscle differentiation and cell fusion to induce hypertrophy [63,64]. Protein quantification in Figure 6C shows RSV action on ERK 1/2 activation during differentiation (pERK1/ERK: RSV 0.1 μM vs DM 24 h, 48 h, 72 h, 96 h p ≤ 0.05; RSV 25 μM vs DM 24 h, 48 h, 72 h p ≤ 0.05) (pERK2/ERK2: RSV 0.1 μM vs DM 48 h, 72 h, 96 h p ≤ 0.05; RSV 25 μM vs DM 48 h, 72 h, 96 h p ≤ 0.05).

AMPK seems to be an essential regulator of muscle cell size maintenance through the control of mTORC1 pathway and can play a major role in the metabolic program that organize muscle plasticity [16-18]. RSV is able to significantly regulate the levels of this important protein. As shown in blot in Figure 6D, RSV caused a significant raise in AMPK protein content during all phases of differentiation (AMPK: RSV 0.1 μM vs DM 24 h, 48 h, 72 h p ≤ 0.05; 96 h p ≤ 0.01; RSV 25 μM vs DM 24 h, 48 h, 72 h p ≤ 0.05). Furthermore, it is important to note how RSV treatment is able to activate AMPK protein also during the last phases of differentiation (pAMPK: RSV 0.1 μM vs DM 96 h p ≤ 0.05; RSV 25 μM vs DM 72 h, 96 h p ≤ 0.05).

Given the essential role in cellular metabolism of AMPK protein, this RSV effect, obtained after stimulation by these doses, assumes a critical relevance.

### Study of the hypertrophic process

**Figure 7:**
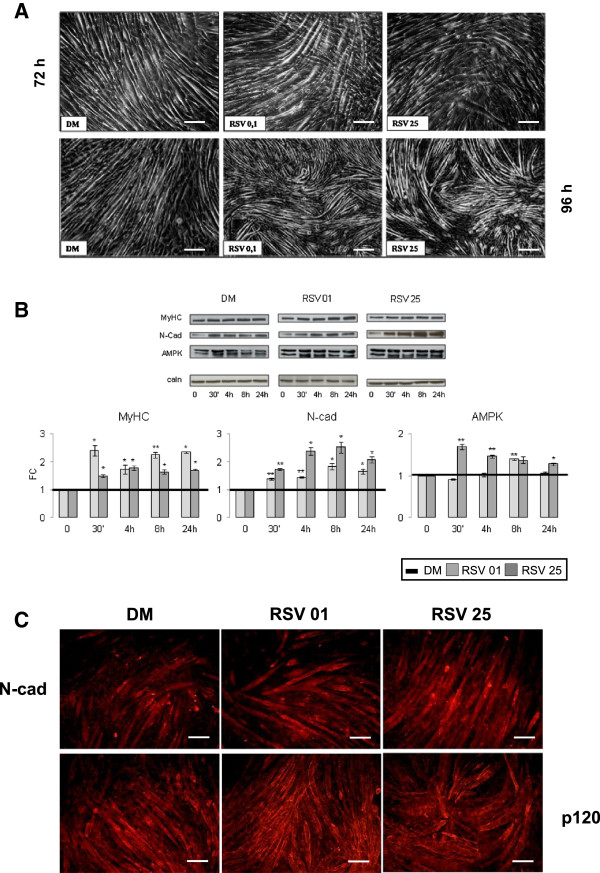
**Protein expression during post-differentiation. A)** Phase contrast images in post-differentiation phase (72 and 96 hours) showed hypertrophy in neo-formed myotubes treated with RSV, in respect with control. Scale bar 200 μm. **B)** Western Blot analysis shows how RSV treatments during post-differentiation phase cause a significant increase in MyHC protein content. Key structural N-Cadherin protein expression is significantly higher than DM. RSV treatments seem to induct a significant rise in AMPK protein content in respect to DM. Significance: * p ≤ 0.05; ** p ≤ 0.01. Representative immunoblots of analyzed proteins are shown. **C)** Immunofluorescence during post-differentiation phase (8 h treatment), with antibodies anti N-Cadherin and p120 Catenin, shows the main increase in size of neo-formed myotubes treated with RSV in respect to DM condition. Scale bar 200 μm.

To confirm RSV involvement in the process of hypertrophy, after 72 hours of differentiation, we performed Western Blot analysis to evaluate protein content after 30 min and 4,8,24 hours of treatment (Figure 1B). Results confirmed the important MyHC protein content increase in RSV stimulated cells (RSV 0.1 μM vs DM 30 min, 4 h, 24 h p ≤ 0.05; RSV 0.1 μM vs DM 8 h p ≤ 0.01; RSV 25 μM vs DM 30 min, 4 h, 8 h, 24 h p ≤ 0.05) (Figure 7B).

Furthermore, during post-differentiation phase, the levels of key structural proteins like N-Cadherin remained high compared to DM control (Figure 7B) (RSV 0.1 μM vs DM 30 min, 4 h p ≤ 0.01; 8 h, 24 h p ≤ 0.05; RSV 25 μM vs DM 30 min p ≤ 0.01; 4 h, 8 h, 24 h p ≤ 0.05).

The same happened for AMPK protein content (RSV 0.1 μM vs DM 8 h p ≤ 0.01; RSV 25 μM vs DM 30 min, 4 h p ≤ 0.01; 24 h p ≤ 0.05) in Figure 7B. In Figure 7A, phase contrast images after 72 and 96 hours of differentiation described morphological features in neo-formed hypertrophic myotubes.

After 8 hours of RSV treatment, Immunofluorescence was performed to study morphological changes of neo-formed myotubes (Figure 7C), monitoring the espression of most important cytoskeletal structural proteins: N-Cadherin and Catenin p120.

**Figure 8:**
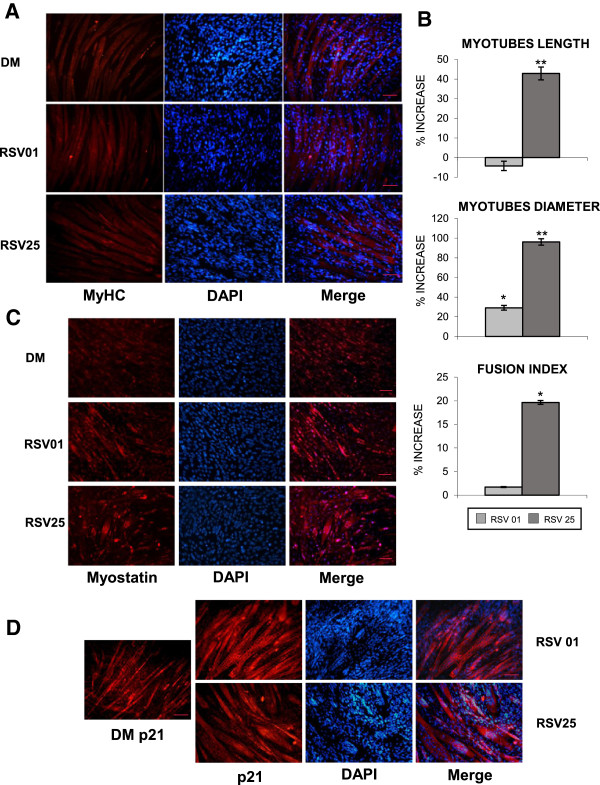
**RSV action on MyHC expression, myotubes dimension, nuclei arrangement in hypertrophy (8 h treatment). A)** Immunofluorescence analysis shows hypertrophic morphological changes in MyHC-positive neo-formed myotubes after RSV treatment. Scale bar 50 μm. **B)** Graphical representation of the significant increment in length, diameter and fusion index of RSV-treated myotubes compared to DM. Significance: *p ≤ 0.05; **p ≤ 0.01. **C)** Myostatin Immunofluorescence images show the nuclei arrangement to form a ring in the central section of myotube, marker of in vitro hypertrophy and maturation, particularly evident in the DAPI nuclei coloration. Scale bar 50 μm. **D)** p21 Immunofluorescence images and DAPI also confirm the nuclei arrangement in neo-formed myotubes after RSV treatments in respect to DM condition. Scale bar 50 μm.

Images in Figure 8, collected after 72 hours of differentiation and 8 hours of RSV treatment, showed the significant increase in size of neo-formed myotubes: increase of length and diameter along with the new central disposition of the nuclei was the evidence of hypertrophy genesis [59,65-67].

To support the RSV involvement in muscle hypertrophy, myotubes dimensions were measured in MyHC images (Figure 8B).

We showed the significant increment in length, diameter and fusion index of RSV-treated myotubes compared to DM condition, in agreement with the evidence that skeletal muscle hypertrophy is characterized by an increase in myofiber size (Figure 8) (Myotubes length: RSV 25 μM vs DM p ≤ 0.01; Myotubes diameter: RSV 0.1 μM vs DM p ≤ 0.05; RSV 25 μM vs DM p ≤ 0.01; Fusion index: RSV 25 μM vs DM p ≤ 0.05).

To assess changes in myotubes nuclear disposition during late phase of differentiation, we performed Immunofluorescence studies (Figure 8A-C-D) using antibodies against MyHC, Myostatin and p21 proteins, which are involved in terminal muscle differentiation. RSV-treated myotubes are characterized by a particular arrangement of the nuclei to form a ring, representing a morphological marker of *in vitro* muscle hypertrophy and maturation [65,67].

## Discussion

Previous studies have demonstrated that the natural polyphenolic phytoalexin Resveratrol possesses various biological, biochemical and physiological actions including anti-inflammatory, anti-oxidant, anti-proliferative, promoting differentiation, and chemo preventive effects in pathological conditions like age-related diseases, cardiovascular diseases, cancer, type 2 diabetes and neurological conditions [26-40].

In skeletal muscle, RSV is involved in muscle metabolism regulation, protein catabolism and function, is able to confer resistance against oxidative stress, injury and death of skeletal muscle cells. Besides, RSV has been shown to improve strength and endurance of skeletal muscle [41-46].

Increasing evidence suggests that RSV has an active role in skeletal muscle differentiation [26,41-46]. However, the mechanisms underlying these RSV-induced adaptations have not been completely elucidated.

In our *in vitro* work, investigating the role of RSV on C2C12 myoblasts growth capacity, we observed its ability to reduce cells proliferation. In support to this result, proliferation rate observed in cell growth curve, elucidates RSV role in the interruption of proliferation. RSV effect was visible not only in the kinetics of cell growth, but also in the morphological analysis: RSV-treated cells lose their originally circular shape to achieve a new, specific, elongate morphology, typical of muscle cell phenotype. It is important to specify that RSV inhibits proliferation without causing cell injury: count and daily observation of C2C12 cells showed the absence of cellular mortality.

Since activation of muscle differentiation program requires irreversible cell cycle withdrawal of C2C12 myoblasts and tissue-specific gene expression, our study was extended investigating the effect of 0.1 and 25 μM RSV on C2C12 myoblasts cell cycle exit. p21 expression is a key event in triggering cell cycle withdrawal and myoblasts differentiation [13,55,56]. During proliferative phase, Western Blot analysis revealed how p21 protein content in DM and RSV (both 0.1 and 25 μM) were super imposable, showing that in these two conditions differentiation process progresses faster than in the growth control condition (GM), wherein the differentiation is only determined by cell contact.

Protein expression of Myf-5 and MyoD transcription factors, myogenic markers already expressed in undifferentiated proliferating myoblasts, was also increased with RSV treatment. In phase contrast and Immunofluorescence images during proliferation phase, the morphological changes mentioned above were clearly visible.

All together, these data support the hypothesis that RSV could regulate myoblasts cell cycle, inducing differentiation process.

The study of differentiation showed how RSV seems to be able to promote the process: 1) inducing the muscle phenotype determination by early expression of MRFs (Myf-5, MyoD and Myogenin), muscle marker proteins (MyHC) and key skeletal structural proteins (N-Cadherin, p120, Alpha Actinin); 2) activating important signaling pathways, including AKT and MAP kinases; 3) causing morphological changes like myoblasts elongation, increase in length and diameter, rise of fusion trend of mono-nucleated myocytes into multinucleated myotubes.

In neo-formed myotubes, RSV seems to maintain hypertrophy process, increasing myotubes size and regulating nuclei arrangement.

Importantly, the present *in vitro* finding may have a potential impact in *in vivo* regulation of protein metabolism. In fact, given RSV action on MRFs and muscle-specific skeletal proteins synthesis joined to the control of AMPK, IGF-1 R [68], AKT [69] and ERK proteins, we may speculate a hypothetical clinical use of this natural polyphenol in conditions of muscle mass damage/hypotrophy. To achieve this aim it is important to further clarify the connection between used RSV doses and observed effects. In fact, several authors indicated that RSV, used in other different doses, shows controversial anti-inflammation and insulin resistance effects [70].

## Conclusions

In summary, our data demonstrate that Resveratrol could control proliferation, start myogenesis process and induce hypertrophy. RSV seems to be able to regulate cell cycle progression, the following cell cycle arrest and early induction of differentiation, through its action on the expression of specific cell cycle regulators, myogenic regulatory factors and muscle-specific structural proteins.

Our *in vitro* studies may constitute novel proof of principle to potential applications of the compound to prevent or reverse muscle impairment by stimulating myogenesis, and emphasize new possible use of RSV to enhance muscle performance.

## Competing interests

The authors declare that they have no competing interests.

## Authors’ contributions

Conceived and designed the experiments: AM, LL, PS, IT. Performed the experiments: AM, PS, IT. Analyzed the data: AM, LL, PS, NM, IT. Wrote the paper: AM, LL, PS, IT. IT had primary responsibility for the final content. All authors read and approved the final manuscript.
